# *SIPA1L3* methylation modifies the benefit of smoking cessation on lung adenocarcinoma survival: an epigenomic–smoking interaction analysis

**DOI:** 10.1002/1878-0261.12482

**Published:** 2019-04-17

**Authors:** Ruyang Zhang, Linjing Lai, Xuesi Dong, Jieyu He, Dongfang You, Chao Chen, Lijuan Lin, Ying Zhu, Hui Huang, Sipeng Shen, Liangmin Wei, Xin Chen, Yichen Guo, Liya Liu, Li Su, Andrea Shafer, Sebastian Moran, Thomas Fleischer, Maria Moksnes Bjaanæs, Anna Karlsson, Maria Planck, Johan Staaf, Åslaug Helland, Manel Esteller, Yongyue Wei, Feng Chen, David C. Christiani

**Affiliations:** ^1^ Department of Biostatistics Center for Global Health School of Public Health Nanjing Medical University China; ^2^ Department of Environmental Health Harvard T.H. Chan School of Public Health Boston MA USA; ^3^ China International Cooperation Center for Environment and Human Health Nanjing Medical University China; ^4^ Department of Epidemiology and Biostatistics School of Public Health Southeast University Nanjing China; ^5^ Department of Biostatistics Harvard T.H. Chan School of Public Health Boston MA USA; ^6^ Department of Preventive Medicine Medical School of Ningbo University China; ^7^ Pulmonary and Critical Care Division Department of Medicine Massachusetts General Hospital Harvard Medical School Boston MA USA; ^8^ Bellvitge Biomedical Research Institute Institucio Catalana de Recerca i Estudis Avançats University of Barcelona Barcelona Spain; ^9^ Department of Cancer Genetics Institute for Cancer Research Oslo University Hospital Norway; ^10^ Division of Oncology and Pathology Department of Clinical Sciences Lund CREATE Health Strategic Center for Translational Cancer Research Lund University Sweden; ^11^ Institute of Clinical Medicine University of Oslo Norway; ^12^ Jiangsu Key Lab of Cancer Biomarkers, Prevention and Treatment Cancer Center Collaborative Innovation Center for Cancer Personalized Medicine Nanjing Medical University China

**Keywords:** DNA methylation, interaction analysis, molecular cancer epidemiology, non‐small‐cell lung cancer, overall survival, smoking cessation

## Abstract

Smoking cessation prolongs survival and decreases mortality of patients with non‐small‐cell lung cancer (NSCLC). In addition, epigenetic alterations of some genes are associated with survival. However, potential interactions between smoking cessation and epigenetics have not been assessed. Here, we conducted an epigenome‐wide interaction analysis between DNA methylation and smoking cessation on NSCLC survival. We used a two‐stage study design to identify DNA methylation–smoking cessation interactions that affect overall survival for early‐stage NSCLC. The discovery phase contained NSCLC patients from Harvard, Spain, Norway, and Sweden. A histology‐stratified Cox proportional hazards model adjusted for age, sex, clinical stage, and study center was used to test DNA methylation–smoking cessation interaction terms. Interactions with false discovery rate‐*q *≤* *0.05 were further confirmed in a validation phase using The Cancer Genome Atlas database. Histology‐specific interactions were identified by stratification analysis in lung adenocarcinoma (LUAD) and lung squamous cell carcinoma (LUSC) patients. We identified one CpG probe (cg02268510_*SIPA*__*1L3*_) that significantly and exclusively modified the effect of smoking cessation on survival in LUAD patients [hazard ratio (HR)_interaction_ = 1.12; 95% confidence interval (CI): 1.07–1.16; *P *=* *4.30 × 10^–7^]. Further, the effect of smoking cessation on early‐stage LUAD survival varied across patients with different methylation levels of cg02268510_*SIPA*__*1L3*_. Smoking cessation only benefited LUAD patients with low methylation (HR **= 0.53; 95% CI: 0.34–0.82; *P *=* *4.61 × 10^–3^) rather than medium or high methylation (HR **= 1.21; 95% CI: 0.86–1.70; *P *=* *0.266) of cg02268510_*SIPA*__*1L3*_. Moreover, there was an antagonistic interaction between elevated methylation of cg02268510_*SIPA*__*1L3*_ and smoking cessation (HR_interaction_ = 2.1835; 95% CI: 1.27–3.74; *P *=* *4.46 × 10^−3^). In summary, smoking cessation benefited survival of LUAD patients with low methylation at cg02268510_*SIPA*__*1L3*_. The results have implications for not only smoking cessation after diagnosis, but also possible methylation‐specific drug targeting.

**Abbreviations**
CI: confidence interval
FDR: false discovery rate
HR: hazard ratio
LUAD: lung adenocarcinoma
LUSC: lung squamous cell carcinoma
NSCLC: non‐small‐cell lung cancer
QC: quality control
SD: standard deviation
TCGA: The Cancer Genome Atlas

## 1 Introduction

Lung cancer is a leading cause of cancer mortality worldwide. In the United States, lung cancer was estimated as likely to account for 154 050 deaths in 2018, or one‐fourth of all cancer deaths (Siegel *et al*., 2017). A large proportion of lung cancer cases are attributed to smoking, a well‐known risk factor (Flanders *et al*., 2003), and smoking cessation prolongs survival and decreases mortality of lung cancer patients (Balduyck *et al*., 2011; Parsons *et al*., 2010). However, the underlying mechanisms of these benefits remain largely unclear (Bhatt *et al*., 2015; Parsons *et al*., 2010).

DNA methylation, a reversible epigenetic modification, regulates gene expression and provides potential cancer biomarkers and therapeutic targets (Egger *et al*., 2004; Feinberg and Tycko, 2004), including for non‐small‐cell lung cancer (NSCLC) (Guo *et al*., 2018; Shen *et al*., 2018; Wei *et al*., 2018). Furthermore, as a potential mechanistic link between cigarette smoking and disease, DNA methylation changes can result from various environmental exposures and may explain part of the association between smoking and cancer recurrence or mortality (Lee and Pausova, 2013; Shui *et al*., 2016).

Progression of complex diseases, such as cancer, results from interactions between clinical, environmental, genetic, and epigenetic factors (Lacombe *et al*., 2016; Mcnerney *et al*., 2017). However, most epigenome‐wide association studies are designed to identify main effects using a standard marginal test (Karlsson *et al*., 2014) while ignoring epigenetic–environment interactions. These traditional mining procedures may reduce the power to identify new epigenomic biomarkers (Slade and Kraft, 2016).

In this study, we hypothesized that epigenetic and smoking cessation interactions may affect NSCLC survival. Epigenome‐wide DNA methylation data composed of four study cohorts containing lung adenocarcinoma (LUAD) and lung squamous cell carcinoma (LUSC) cases were used for discovery, and the findings were independently validated in The Cancer Genome Atlas (TCGA) data.

## 2 Materials and methods

### 2.1 Study population

Early‐stage (stage I–II) LUAD and LUSC patients who were former or current smokers were included in the study. Never smokers were defined as those who smoked ≤ 100 cigarettes over a lifetime. Current smokers were defined as those who were smoking within 1 year of diagnosis. Former smokers were defined as smokers who quit > 1 year before diagnosis or interview (Suk *et al*., 2006). We encoded the variable smoking cessation as ‘yes’ for former smokers and ‘no’ for current smokers. Data were harmonized from five international study centers, which have been previously described (Guo *et al*., 2018; Shen *et al*., 2018; Wei *et al*., 2018; Zhang *et al*., 2019). All patients provided written informed consent, and the study methodologies conformed to the standards set by the Declaration of Helsinki and received approval by its respective institutional review board.

#### 2.1.1 Harvard

The Harvard Lung Cancer Study cohort was described previously (Suk *et al*., 2006). Briefly, all patients were recruited at Massachusetts General Hospital (MGH) from 1992 to present and had newly diagnosed, histologically confirmed primary NSCLC. We included 133 early‐stage LUAD and LUSC patients who were former or current smokers for the current study. DNA was extracted from tumor specimens that were evaluated by an MGH pathologist for amount (tumor cellularity > 70%) and quality of tumor cells and histologically classified using World Health Organization criteria. The study protocol was approved by the Institutional Review Boards at Harvard School of Public Health and MGH.

#### 2.1.2 Spain

The Spain study population was reported previously (Sandoval *et al*., 2013) and included 196 LUAD and LUSC patients recruited at eight subcenters from 1991 to 2009. In brief, tumor DNA was extracted from fresh‐frozen tumor specimens that were collected by surgical resection, and the median clinical follow‐up was 7.2 years. The study was approved by the Bellvitge Biomedical Research Institute institutional review board.

#### 2.1.3 Norway

The Norway cohort consisted of 116 LUAD patients with operable lung cancer tumors who were seen at Oslo University Hospital, Rikshospitalet, Norway, in 2006–2011 (Bjaanæs *et al*., 2016). Tumor tissues obtained during surgery were snap‐frozen in liquid nitrogen and stored at −80 °C until DNA isolation. The project was approved by Oslo University institutional review board and regional ethics committee (S‐05307).

#### 2.1.4 Sweden

The Sweden cohort included 85 LUAD and LUSC patients. Tumor DNA was collected from early‐stage lung cancer patients who underwent an operation at the Skane University Hospital, Lund, Sweden (Karlsson *et al*., 2014). The study was approved by the Regional Ethical Review Board in Lund, Sweden (Registration no. 2004/762 and 2008/702).

#### 2.1.5 TCGA

The TCGA database contains 562 early‐stage LUAD and LUSC patients who have full information of survival time and covariates. Level 1 HumanMethylation450 DNA methylation data (image data) for each patient were downloaded on October 1, 2015.

### 2.2 Quality control procedures

DNA methylation was profiled using Infinium HumanMethylation450 BeadChips (Illumina Inc., San Diego, CA, USA) for all patients. Raw image data were imported into GenomeStudio Methylation Module V1.8 (Illumina Inc.) to calculate methylation signals and to perform normalization, background subtraction, and quality control (QC). Beta values, which range from 0% (unmethylated) to 100% (methylated), were used to measure methylation level of each probe. Unqualified probes were excluded if they met any one of the following QC criteria: (a) failed detection (*P *>* *0.05) in > 5% samples; (b) coefficient of variance of < 5%; (c) methylated or unmethylated in all samples; (d) common single nucleotide polymorphisms located in probe sequence or 10‐bp flanking regions; (e) cross‐reactive or cross‐hybridizing probes (Chen *et al*., 2013); or (f) did not pass QC in all centers. Samples with > 5% undetectable probes were excluded. Methylation signals were further processed for quantile normalization, design bias correction for type I and II probes, and batch effect adjustment using *ComBat* correction (Marabita *et al*., 2013). We performed QC procedures above in each center separately and then merged all data together before association analysis. Details of QC processes are described in Fig. S1.

### 2.3 Gene expression data

Expression and mRNA sequencing data were available for 281 LUAD and 277 LUSC patients of the TCGA dataset (Table S1). TCGA mRNA sequencing data processing and QC were done by the TCGA workgroup. Raw counts were normalized using RNA sequencing by expectation maximization. Level 3 gene quantification data were downloaded from the TCGA data portal (https://tcga-data.nci.nih.gov; now hosted at https://portal.gdc.cancer.gov) and were further checked for quality. Gene expression data were extracted and log2‐transformed before analysis.

### 2.4 Epigenome‐wide DNA methylation–smoking cessation interaction analysis

**Figure 1:**
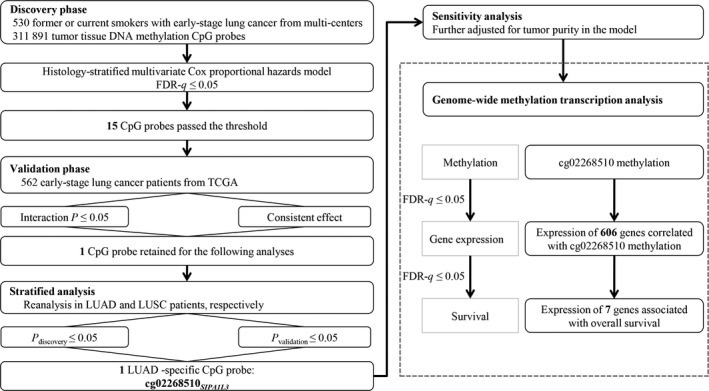
Flowchart of study design and statistical analyses.

Analysis flow is described in Fig. 1. Patients from the first four study centers (Harvard, Spain, Norway, and Sweden) were assigned into the discovery phase. A histology‐stratified Cox proportional hazards model was used to test the interaction item, which was the interaction effect between DNA methylation of each CpG probe and smoking cessation (CpG probe × smoking cessation) on overall survival. The model was adjusted for age, sex, smoking cessation, clinical stage, and study center. Hazard ratio (HR) and 95% confidence interval (CI) were described per 1% methylation increment. Multiple testing corrections were performed using the false discovery rate method (FDR, measured by FDR‐*q* value) by the Benjamini–Hochberg procedure. CpG probes with interaction FDR‐*q *≤* *0.05 were replicated in the validation phase using the TCGA dataset. Robustly significant probes were retained if they met all criteria: (a) interaction *P *≤* *0.05 in the validation phase; and (b) consistent effect direction in both discovery and validation phases. We performed stratified analysis for robustly significant CpG probes in LUAD and LUSC patients. Finally, CpG probes with a significant interaction with smoking cessation in both phases were identified as histology‐specific probes.

### 2.5 Sensitivity analysis for significant CpG probes

Due to the complex tumor microenvironment—including noncancerous components, which might alter analysis of tumor samples (Aran *et al*., 2015)—we assessed tumor purity with InfiniumPurify (Zhang *et al*., 2015) using methylation array data from TCGA samples. Tumor purity was included as an additional covariate in the Cox regression model for sensitivity analysis.

### 2.6 Genome‐wide methylation transcription analysis

For robustly significant histology‐specific prognostic CpG probes, we also performed genome‐wide methylation transcription analysis using mRNA sequencing data from TCGA. The correlation between DNA methylation and gene expression was tested using a linear regression model adjusted for the same covariates mentioned above. Association with FDR‐*q *≤* *0.05 was considered significant. Additionally, we tested the association between gene expression and overall survival using a Cox proportional hazards model adjusted for the same covariates. Genes involved in significant associations with both methylation and NSCLC survival were filtered.

### 2.7 Statistical analysis

Continuous variables were summarized as mean ± standard deviation (SD), and categorized variables were described by frequency (*n*) and proportion (%). Kaplan–Meier survival curves were used to compare survival difference among subgroups. Statistical analyses were performed using r version 3.4.4 (The R Foundation for Statistical Computing).

## 3 Results

After QC, epigenome‐wide DNA methylation data including 311 891 CpG sites from 1092 tumor samples of early‐stage (stage I–II) NSCLC patients were retained. There were 530 patients (*N*_LUAD _= 413 and *N*_LUSC _= 117) in the discovery phase and 562 patients (*N*_LUAD _= 285 and *N*_LUSC _= 277) in the validation phase. Table 1 details demographic and clinical information for the study population. There were 37% and 27% current smokers in the discovery and validation phases, respectively.

**Table 1:** Demographic and clinical characteristics of former and current smokers with early‐stage NSCLC in five study centers

Variable	Discovery phase					Validation phase	Combined data
	Harvard (*N *=* *133)	Spain^a^ (*N *=* *196)	Norway (*N *=* *116)	Sweden (*N *=* *85)	Discovery: All (*N *=* *530)	TCGA (*N *=* *562)	Overall (*N *=* *1092)
Age (years), mean ± SD	68.04 ± 9.39	65.69 ± 10.41	64.89 ± 8.94	65.65 ± 9.69	66.10 ± 9.78	66.55 ± 9.39	66.33 ± 9.58
Sex, *n* (%)							
Female	56 (42.11)	78 (39.80)	59 (50.86)	42 (49.41)	235 (44.34)	217 (38.61)	452 (41.39)
Male	77 (57.89)	118 (60.20)	57 (49.14)	43 (50.59)	295 (55.66)	345 (61.39)	640 (58.61)
Smoking cessation, *n* (%)							*
No	52 (39.10)	71 (36.22)	42 (36.21)	31 (36.47)	196 (36.98)	168 (26.89)	364 (33.33)
Yes	81 (60.90)	120 (61.22)	74 (63.79)	54 (63.53)	329 (62.08)	376 (66.90)	705 (64.56)
Unknown	0	5	0	0	5	18	23
Race, *n* (%)							
White	133 (100)	196 (100)	116 (100)	85 (100)	530 (100)	444 (79.00)	974 (89.19)
Black or African American	0	0	0	0	0	54 (9.61)	54 (4.95)
Asian	0	0	0	0	0	5 (0.89)	5 (0.46)
Unknown	0	0	0	0	0	59	59
TNM stage, *n* (%)							*
I	87 (65.41)	160 (81.63)	80 (68.97)	79 (92.94)	406 (76.60)	358 (63.70)	764 (69.96)
II	46 (34.59)	36 (18.37)	36 (31.03)	6 (7.06)	124 (23.40)	204 (36.30)	328 (30.04)
Histology, *n* (%)							*
LUAD	79 (59.40)	155 (79.08)	116 (100)	63 (74.12)	413 (77.92)	285 (50.71)	698 (63.92)
LUSC	54 (40.60)	41 (20.92)	0	22 (25.88)	117 (22.08)	277 (49.29)	394 (36.08)
Chemotherapy, *n* (%)							*
No	125 (93.98)	157 (80.10)	86 (74.14)	51 (60.00)	419 (79.06)	178 (31.67)	597 (54.67)
Yes	8 (6.02)	15 (7.65)	30 (25.86)	6 (7.06)	59 (11.13)	53 (9.43)	112 (10.26)
Unknown	0	24	0	28	52	331	383
Radiotherapy, *n* (%)							
No	115 (86.47)	161 (82.14)	115 (99.14)	57 (67.06)	448 (84.53)	220 (39.15)	668 (61.17)
Yes	18 (13.53)	11 (5.61)	1 (0.86)	0	30 (5.66)	11 (1.96)	41 (3.75)
Unknown	0	24	0	28	52	331	383
Adjuvant therapy, *n* (%)							*
No	110 (82.71)	148 (75.51)	85 (73.28)	51 (60.00)	394 (74.34)	172 (30.60)	556 (51.83)
Yes	23 (17.29)	24 (12.24)	31 (26.72)	6 (7.06)	84 (15.85)	59 (10.50)	143 (13.10)
Unknown	0	24	0	28	52	331	383
Survival year							
Median (95% CI)	6.44 (4.93–7.44)	6.53 (5.06–8.70)	7.34 (6.70–7.98)^c^	5.89 (4.18–8.82)	7.12 (6.23–7.95)	4.54 (3.69–5.41)	6.23 (5.55–7.20)
Censored rate^b^, %	14.29	54.59	68.97	40.00	45.30	76.16	61.17

a Spain is a collaborative study center, containing samples from Spain, Italy, the UK, France, and the United States.

b Censored rate is the proportion of samples lost to follow‐up or alive at the study end.

c Restricted mean survival time is provided because median was not available.

* Statistically significant difference (*P *≤* *0.05) was observed between combined discovery set and validation set (TCGA).

**Figure 2:**
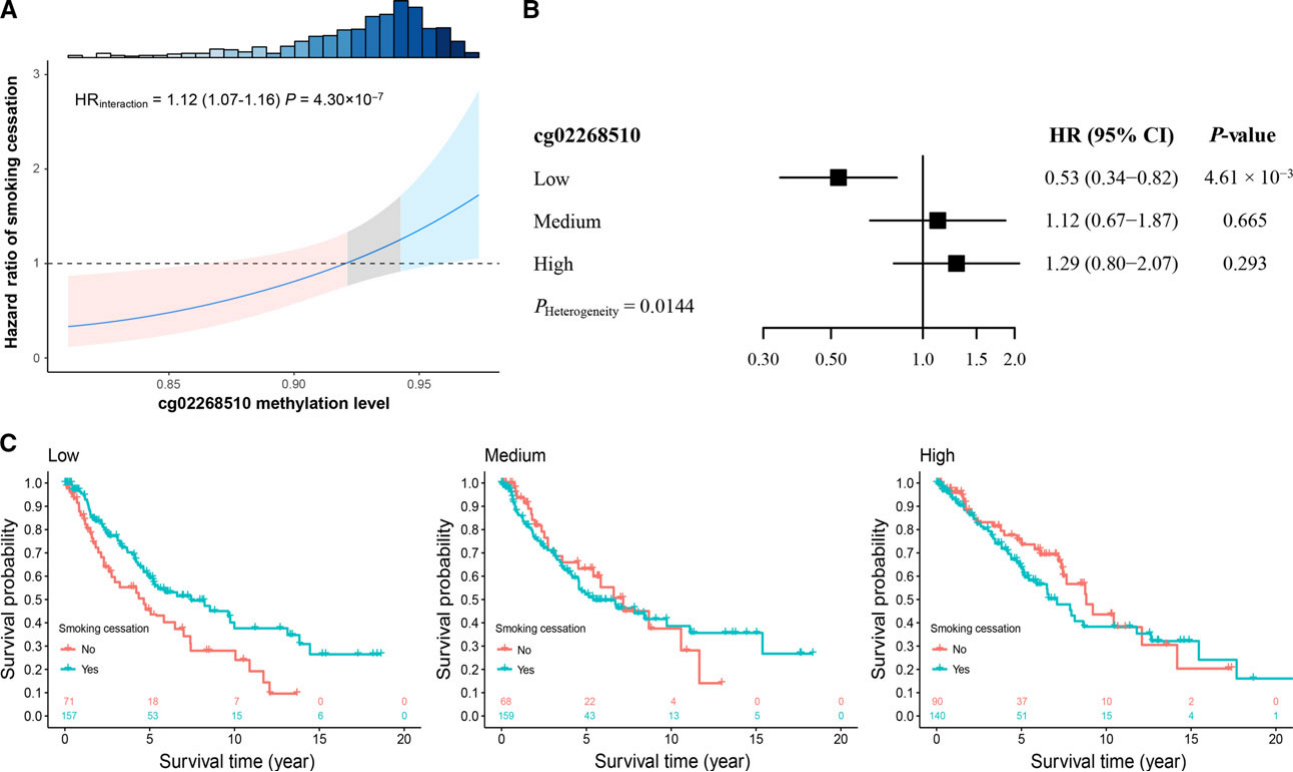
DNA methylation and smoking cessation interaction on survival of LUAD patients. (A) HR of smoking cessation estimated based on methylation level of cg02268510. The shallow area represents 95% CI, with red, gray and blue areas indicating low, medium and high methylation, respectively. Histogram on the top shows the distribution of methylation. (B) Forest plots of the effects of smoking cessation among combined LUAD populations with low, medium, or high methylation of cg02268510. *P*_heterogeneity_ was used to evaluate heterogeneity of HRs across groups. (C) Kaplan–Meier survival curves of current and former smokers among LUAD patients with varying methylation levels.

In the discovery phase, 15 methylation–smoking cessation interactions were identified with FDR‐*q *≤* *0.05 (Fig. S2A), and the Manhattan plot also showed the results for main effect additionally (Fig. S2B). Only 1 interaction remained statistically significant in the validation phase under the most stringent criteria (Table S2). This site, cg02268510, is located in signal‐induced proliferation‐associated 1‐like 3 (*SIPA1L3*). Further histology‐stratified analysis showed that cg02268510_*SIPA1L3*_ is a LUAD‐specific CpG probe that interacts with smoking cessation to affect patient survival in the discovery phase (HR_interaction_ = 1.10; 95% CI: 1.05–1.16; *P *=* *2.95 × 10^–5^), the validation phase (HR_interaction_ = 1.17; 95% CI: 1.02–1.35; *P *=* *0.0255), and the combined data (HR_interaction_ = 1.12; 95% CI: 1.07–1.16; *P *=* *4.30 × 10^–7^). Moreover, fixed‐effect meta‐analysis of five centers also remained significant (HR_interaction_ = 1.09; 95% CI: 1.05–1.13; *P *=* *6.66 × 10^–6^; Fig. S3). As presented in Fig. 2A, with decreased methylation level of cg02268510_*SIPA1L3*_, there was an elevated benefit effect size of smoking cessation on LUAD survival. Thus, there was a modification effect of cg02268510_*SIPA1L3*_ on the association between smoking cessation and survival.

After including tumor purity as an additional covariate in sensitivity analysis, DNA methylation at cg02268510_*SIPA1L3*_ retained a significant interaction with smoking cessation on LUAD survival (HR_interaction_ **= 1.18; 95% CI: 1.02–1.36; *P *=* *0.024). The interaction *P*‐value was still significant but slightly inflated due to (a) the smaller sample size (51% of original) of the sensitivity analysis, which was only performed in TCGA; and (b) low tumor purity (~ 60%) for NSCLC samples in TCGA due to mixed cell types (Zheng *et al*., 2017).

To better illustrate the interaction pattern between DNA methylation and smoking cessation, patients were categorized into low, medium, and high groups based on tertiles of cg02268510_*SIPA1L3*_ methylation. The effect of smoking cessation varied across LUAD patients with different DNA methylation levels. Smoking cessation only benefited LUAD patients with low methylation of cg02268510_*SIPA1L3*_ (HR_low_ **= 0.53; 95% CI: 0.34–0.82; *P *=* *4.61 × 10^–3^). However, there was no significant association between smoking cessation and survival in LUAD patients with medium–high methylation of cg02268510_*SIPA1L3*_ (HR_medium_ **= 1.12; 95% CI: 0.67–1.87; *P *=* *0.665; HR_high_ **= 1.29; 95% CI: 0.80–2.07; *P *=* *0.293; HR_medium–high_ **= 1.21; 95% CI: 0.86–1.70; *P *=* *0.266). We observed significant heterogeneity of smoking cessation effect across the three groups (*P *=* *0.014; Fig. 2B), and Kaplan–Meier curves confirmed these results (Fig. 2C).

**Figure 3:**
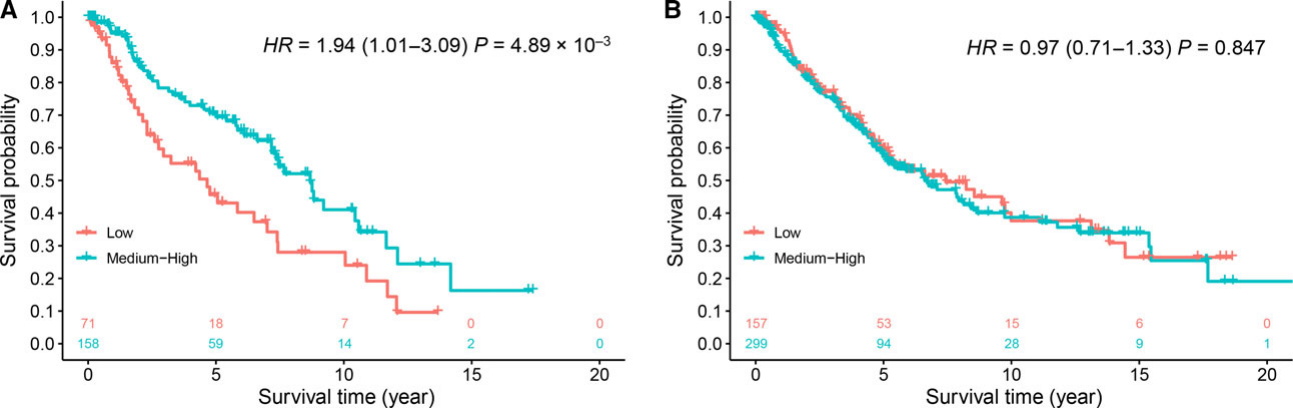
Kaplan–Meier survival curves of LUAD patients categorized into low and medium–high methylation groups according to tertiles in different smoking cessation groups: (A) current smokers (No) and (B) former smokers (Yes). HR, 95% CI, and *P*‐value were derived from the Cox proportional hazards model adjusted for age, sex, clinical stage, and study center.

These results also indicated that LUAD patients who did not quit smoking (current smokers) had the poorest prognosis if their methylation of cg02268510_*SIPA1L3*_ was in a low level. So we combined the medium and high methylation groups and performed further analysis. Current smokers in the low methylation group had 1.94 times the mortality risk compared with the medium or high methylation group (Fig. 3A), but there was no statistically significant difference between groups for former smokers (Fig. 3B). The results also indicated that smoking cessation was quite urgent for LUAD patients with low methylation of cg02268510_*SIPA1L3*_.

In addition, we evaluated the joint effect of CpG methylation level (medium–high vs low) and smoking cessation (Yes vs No) on LUAD survival (Table 2). We used the poorest‐prognosis group (current smokers with low methylation) as the reference to evaluate effect of elevated methylation level, smoking cessation, and their interaction. In the combined dataset, the effect of smoking cessation was HR =* *0.5506 (95% CI: 0.36–0.84; *P *=* *5.62 × 10^−3^) and the effect of medium–high methylation of cg02268510_*SIPA1L3*_ was HR =* *0.5214 (95% CI: 0.34–0.81; *P *=* *3.48 × 10^−3^). However, the joint effect was HR =* *0.6268 (95% CI: 0.43–0.92; *P *=* *1.84 × 10^−2^), which was greater than the product of the two individual protective effects (0.5506 × 0.5214 = 0.2871). The joint effect of two protective factors was less protective than expected, indicating an antagonistic interaction between elevated methylation of cg02268510_*SIPA1L3*_ and smoking cessation (HR_interaction_ = 2.1835; 95% CI: 1.27–3.74; *P *=* *4.46 × 10^−3^).

**Table 2:** Joint effect and interaction of elevated methylation and smoking cessation on the prognosis of early‐stage LUAD

Effect type^a^	Medium–high methylation^b^	Smoking cessation	Number	HR (95% CI)^a^	*P* a
	No	No	71	Ref.	
Main effect_1_	No	Yes	157	0.5506 (0.3609, 0.8400)	5.62 × 10^−3^
Main effect_2_	Yes	No	158	0.5214 (0.3369, 0.8070)	3.48 × 10^−3^
Joint effect	Yes	Yes	299	0.6268 (0.4251, 0.9243)	1.84 × 10^−2^
Interaction^c^				2.1835 (1.2747, 3.7401)	4.46 × 10^−3^

a Patients were categorized into two groups (medium–high vs low) by tertiles of cg02268510_*SIPA1L3*_ methylation level.

b Main effects of elevated methylation and smoking cessation and their joint effect and interaction were derived from the Cox proportional hazards model adjusted for covariates.

c Interaction = Joint effect ÷ (main effect_1_ × main effect_2_). 2.1835 = 0.6268 ÷ (0.5506 × 0.5214).

A growing body of research has reported potential associations of DNA methylation with age and smoking (Fraga and Esteller, 2007; Wan *et al*., 2012; Zaghlool *et al*., 2015). Therefore, we also tested the association between methylation of cg02268510_*SIPA1L3*_ and age, as well as smoking‐related variables: pack‐year of smoking, years of smoking, and years of smoking cessation using a linear regression model adjusted for age, sex, clinical stage, and study centers. Smoking‐related characteristics of former and current smokers in early‐stage LUAD are described in Table S3. There was no significant association between methylation of cg02268510_*SIPA1L3*_ and age (β = −0.01; *P *=* *0.521) or years of smoking (β = 0.03; *P *=* *0.210), but pack‐year of smoking (β = 0.02; *P *=* *3.42 × 10^−3^) as well as years of smoking cessation (β = −0.06; *P *=* *5.08 × 10^−3^) in former smoker LUAD patients (Fig. S4).

**Figure 4:**
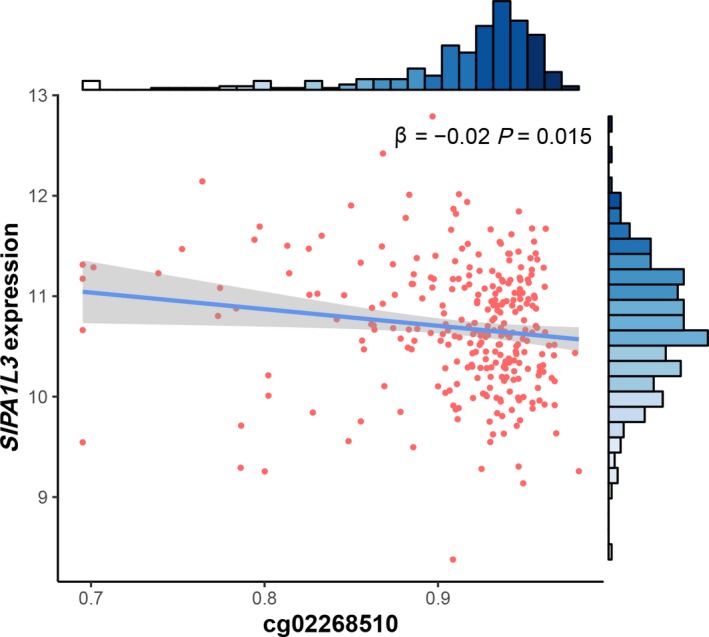
Association between DNA methylation of cg02268510 and expression of corresponding gene *SIPA1L3*. The β coefficient and *P*‐value were based on linear regression analysis adjusted for age, sex, smoking status, and clinical stage. Gene expression was log2‐transformed before analysis.

Further, because cg02268510_*SIPA1L3*_ maps to *SIPA1L3*, the association between cg02268510_*SIPA1L3*_ and *SIPA1L3* expression was evaluated using the TCGA dataset. We observed a significant association between cg02268510_*SIPA1L3*_ and *SIPA1L3* expression (β = −0.02; *P *=* *0.015) in LUAD patients (Fig. 4), indicating that cg02268510_*SIPA1L3*_ *cis*‐regulates gene expression. Moreover, genome‐wide methylation transcription analysis revealed that expression of 633 genes was significantly correlated with methylation level of cg02268510_*SIPA1L3*_ (Fig. S5A). Among them, expression of only seven genes was significantly associated with overall survival: growth arrest and DNA damage‐inducible gamma (*GADD45G*), maturin (*MTURN*), *TMEM200B*,* RGS20*, RELT‐like 1 (*RELL1*), *PGM2,* and receptor‐interacting serine/threonine kinase 2 (*RIPK2*; Fig. S5B–H).

## 4 Discussion

In this study, we systematically evaluated all pairwise DNA methylation–smoking cessation interactions on an epigenome‐wide scale and further confirmed these interactions in an independent population. To our knowledge, this is the first study with a large sample size to investigate interactions between DNA methylation and smoking behavior on lung cancer survival, and it provides new evidence to account for the missing heritability of complex diseases (Trerotola *et al*., 2015). Our results show that the effect of smoking cessation on early‐stage LUAD patient survival varies with methylation level of cg02268510_*SIPA1L3*_. Smoking cessation only benefits LUAD patients with low methylation, rather than medium or high methylation, of cg02268510_*SIPA1L3*_. Further, there is an antagonistic interaction between elevated methylation of cg02268510_*SIPA1L3*_ and smoking cessation.

We found that in LUAD patients with low methylation of cg02268510_*SIPA1L3*_, current smokers with more accumulative exposure had worse survival than former smokers. However, for a population with medium–high methylation, the prognosis of current smokers was similar to that of former smokers. The effect of smoking cessation is therefore modified by DNA methylation level, indicating opportunities for epi‐drug intervention due to the inherent reversibility of epigenetic events (Wright, 2013).

Up to 50% of lung cancer patients are estimated to keep smoking after diagnosis or to frequently relapse after smoking cessation (Park *et al*., 2012; Walker *et al*., 2006). Our results indicated that smoking cessation was urgent especially for LUAD patients with low methylation of cg02268510_*SIPA1L3*_. On the other hand, reduced methylation of cg02268510_*SIPA1L3*_ might strengthen the protective effect of smoking cessation on survival.

Many studies have reported significant associations between smoking cessation and overall survival (Koshiaris *et al*., 2017; Nia *et al*., 2005), while other studies have reported negative results (Baser *et al*., 2006; Parsons *et al*., 2010). Based on our interaction analysis, we suspected that epigenetic modifications might account for this inconsistent phenomenon. Because the effect of smoking cessation varies across populations with different methylation levels of cg02268510_*SIPA1L3*_, the effect could be neutralized in a population of patients with mixed cg02268510_*SIPA1L3*_ methylation levels. Thus, the traditional marginal test for association between smoking cessation and cancer survival inherently loses statistical power to report significant findings due to complex association patterns.

**Figure 5:**
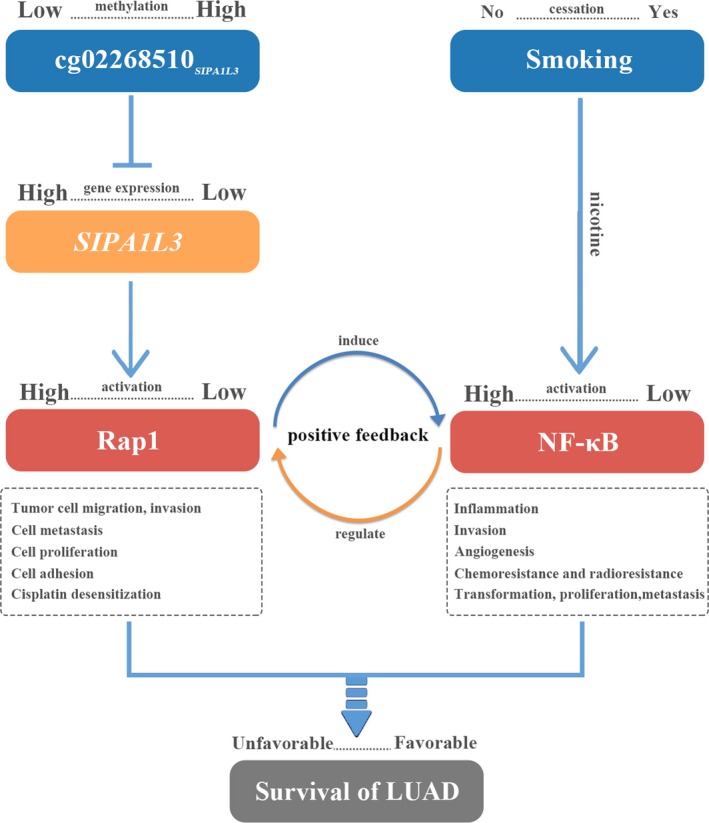
Diagram for pathway of DNA methylation–smoking cessation interaction effect on survival for LUAD patients.

*SIPA1L3*, the gene in which cg02268510 is located, encodes GTPase‐activating proteins (GAPs) specific for the GTP‐binding protein Ras‐associated protein‐1 (RAP1), which is implicated in regulation of cell adhesion, cell polarity, and cytoskeletal organization (Kooistra *et al*., 2007). *SIPA1L3* is a member of the SPA1 family of RapGAPs, which play a crucial role in spatiotemporal control of Rap1 activation in cells (Mochizuki *et al*., 2001). Rap1 plays many roles during cell invasion and metastasis in different cancers (Zhang *et al*., 2017). Additionally, overexpression of RAP1 may desensitize NSCLC cells to cisplatin, a first‐line drug to treat NSCLC (Besse *et al*., 2014). Our results suggest that low methylation at cg02268510_*SIPA1L3*_ might promote *SIPA1L3* expression, further leading to Rap1 activation and resulting in poor prognosis (Fig. 5).

Many of the deleterious effects of smoking are due to induction of inflammatory responses that contribute to lung cancer progression (Crusz and Balkwill, 2015; Walser *et al*., 2008). *In vitro* experiments in human umbilical vein endothelial cells demonstrate that nicotine stimulates cellular inflammatory responses by activating the NF‐κB transcription factor axis by a second messenger pathway (Ueno *et al*., 2006). Activation of NF‐κB, one of the most investigated transcription factors, controls multiple cellular processes in cancer, including inflammation, transformation, proliferation, angiogenesis, invasion, metastasis, chemoresistance, and radioresistance (Chaturvedi *et al*., 2010). Nicotine protects NSCLC cells against chemotherapy‐induced apoptosis and serum deprivation‐induced apoptosis through NF‐κB, and NF‐κB activity is also directly stimulated by nicotine (Anto *et al*., 2002; Tsurutani *et al*., 2005). Therefore, for current smokers, nicotine in tobacco stimulates activation of NF‐κB, induces inflammatory responses, and is relevant to poor patient prognosis (Fig. 5).

Moreover, Rap1 is an essential modulator of NF‐κB‐mediated pathways. NF‐κB is induced by ectopic expression of Rap1, whereas its activity is inhibited by Rap1 depletion (Teo *et al*., 2010). Furthermore, levels of Rap1 are positively regulated by NF‐κB, and human breast cancers with NF‐κB hyperactivity show elevated levels of cytoplasmic Rap1 (Teo *et al*., 2010). Thus, positive feedback mechanisms might exist between Rap1 expression and NF‐κB activation (Fig. 5). In terms of cg02268510_*SIPA1L3*_ and smoking cessation interaction, keeping smoking was associated with poor prognosis only in LUAD patients with low methylation, rather than medium or high methylation, possibly because high activation of both Rap1 and NF‐κB may only occur in patients with low methylation.

We also found that methylation level of cg02268510_*SIPA1L3*_ increased along with long pack‐year of smoking, but decreased with long years of smoking cessation. As presented in Fig. 5, low methylation and keeping smoking resulted in the worst prognosis, which might be due to the positive feedback in Rap1 and NF‐κB. However, methylation of cg02268510_*SIPA1L3*_ increased with the cumulative amount of smoking. But, high methylation of cg02268510_*SIPA1L3*_ resulted in low *SIPA1L3* expression that was hard to active Rap1 and NF‐κB, and then might weaken the harmful effect of smoking, which also indicated an antagonistic effect. It is implied that there might be a self‐protective mechanism in the human body that prevents the body from receiving excessive damage from exposure. As reported, smoking increases reactive oxygen species (ROS) production and is a significant source of oxidative stress (Athanasios *et al*., 2013), but *in vivo*, there is a variety of antioxidant defense mechanisms existed to counteract the detrimental effects of ROS by regulating the production of free radicals and their metabolites (Deponte, 2013; He *et al*., 2017). It may be an adaptive defense mechanism to counteract the increased ROS production that superoxide dismutase enzyme levels in blood and salivary were increased in smokers (Jenifer *et al*., 2015). Moreover, a previous study has found that activation of Rap1 serves to attenuate ROS production (Remans *et al*., 2004) and there is a potential interrelationship between Rap1, ROS, and NF‐κB activation (Moon *et al*., 2011). But further functional studies are warranted to elucidate the mechanism of cg02268510_*SIPA1L3*_ and smoking cessation interaction on LUAD survival.

Meanwhile, we observed that some genes trans‐regulated by cg02268510_*SIPA1L3*_ are involved in DNA damage response and cell growth (*GADD45G*) (Guo *et al*., 2013), immune cell functions (*MTURN*) (Sun *et al*., 2014), tumor cell migration [regulator of G protein signaling 20 (*RGS20*)] (Yang *et al*., 2016), apoptosis (*RELL1* and *RIPK2*) (Chin *et al*., 2002; Cusick *et al*., 2010), and innate and adaptive immunity (*RIPK2*) (Jaafar *et al*., 2018).

*GADD45G* is a member of the GADD45 family, which plays an essential role in cellular stress response, survival, senescence, and apoptosis regulation (Liebermann *et al*., 2011). *GADD45G* has been reported to be a tumor suppressor in multiple cancer types and can inhibit cell growth and induce apoptosis (Ying *et al*., 2005). Patients with high *GADD45G* expression had a better prognosis in our study. *MTURN* is a neural progenitor differentiation regulator homolog. 12‐O‐tetradecanoylphorbol‐13‐acetate (TPA) is an effective cancer therapeutic reagent for myelocytic leukemia patients (Han *et al*., 1998), and *MTURN* is TPA‐responsive and may promote both leukemic and normal megakaryocyte differentiation (Sun *et al*., 2014). Indeed, differentiation therapy by forced differentiation of cancer cells has been successful in curing acute promyeloid leukemia (Chen *et al*., 2011). Similarly, LUAD patients with high *MTURN* expression had favorable survival in our study. *RGS20* is suggested to promote cellular characteristics that contribute to metastasis, including enhanced cell aggregation, motility, and invasion. Selective inhibition of RGS20 expression may represent an alternative means to suppress metastasis (Yang *et al*., 2016). Its high expression is significantly associated with progression and prognosis of triple‐negative breast cancer (Li *et al*., 2017). Additionally, our study showed similar results in LUAD patients. Though there is a lack of explicit evidence of relevance between these genes and smoking, what we found may inspire functional studies of these potential genes and further help to complete a picture of the mechanism pathway of cg02268510_*SIPA1L3*_ and smoking cessation interaction on LUAD survival.

Our study has some significant strengths. First, this is the first study to investigate the interaction between DNA methylation and smoking cessation on lung cancer survival on an epigenome‐wide scale, which provides new evidence to account for the missing heritability of complex diseases (Trerotola *et al*., 2015). Second, the two‐stage study design we used to exhaustively search for interactions, as well as the sensitivity analysis, is quite conservative in controlling for false positives. Third, our study included a large sample size to analyze DNA methylation–smoking cessation interactions of early‐stage NSCLC prognosis, providing an opportunity to identify complex associations with small–medium effect size.

Despite the strengths of our study, we acknowledge some limitations. First, data measured categorical smoking cessation rather than smoking pack‐years, which may render less power in the study. Second, smoking cessation was collected at the time of diagnosis and was not reassessed during follow‐up. Previous studies have found that ‘former smokers’ might more accurately represent a mixed exposure status, since quitters are more likely to relapse (Hughes *et al*., 2004; Walker *et al*., 2006). Thus, we likely underestimated the benefits of smoking cessation. Third, the association between cg02268510_*SIPA1L3*_ and expression of several genes requires more biological evidence, though methylation is believed to play a crucial role in regulating gene expression (Bird, 2007) and further influence disease gene function (Schübeler, 2015), cell differentiation, or reprogramming (Khavari *et al*., 2010). Thus, functional experiments are warranted to confirm these associations, so our findings should be biologically interpreted with caution thus far. In addition, our study consisted mainly of a Caucasian population (89.19%), since TCGA data contained only ~ 10% non‐Caucasian samples. Our results should therefore be translated with caution for other populations. Lastly, the censored rate of survival time for the TCGA population is relatively high, since early‐stage NSCLC patients need longer follow‐up time. Thus, the validation phase using TCGA population had low statistical power. However, we still successfully replicated one significant interaction, indicating a quite conservative and robust result (Leung *et al*., 1997; Watt *et al*., 1996).

## 5 Conclusion

This epigenome‐wide DNA methylation–smoking cessation interaction analysis of early‐stage NSCLC identified one LUAD‐specific CpG probe, cg02268510_*SIPA1L3*_ *,* which could significantly modify effects of smoking cessation on lung cancer survival. Smoking cessation benefited survival of LUAD patients with low methylation at cg02268510_*SIPA1L3*_. These results have implications for not only smoking cessation after diagnosis, but also possible methylation‐specific drug targeting.

## Conflict of interest

The authors have no conflicts to declare.

## Author contributions

RZ, LLai, YW, and FC contributed to the study design. RZ, SM, TF, MMB, AK, MP, JS, AH, ME, AS, and LS contributed to data collection. RZ, LLai, and YW performed statistical analysis and interpretation and drafted the manuscript. XD, JH, DY, CC, LLin, YZ, HH, SS, LW, XC, YG, and LLiu revised the manuscript. All authors contributed to critical revision of the manuscript and approved its final version. Financial support and study supervision were provided by RZ, FC, and DCC.

## Supporting information


**Fig. S1.** Quality control processes for DNA methylation chip data.
**Fig. S2.** Manhattan plot of DNA methylation–smoking cessation interaction *P*‐values (A) and main effect *P*‐values (B) derived from histology‐stratified Cox proportional hazards model in the discovery phase.
**Fig. S3.** Fixed‐effect meta‐analysis of interaction between DNA methylation of cg02268510 and smoking cessation for LUAD patients from five centers.
**Fig. S4.** Linear regression analysis between methylation of cg02268510 and age (A) as well as smoking‐related variables: pack‐year of smoking (B), year of smoking (C), and year of smoking cessation (D), adjusted for age, sex, smoking status, clinical stage, and study center.
**Fig. S5.** Genome‐wide methylation transcription analysis results from the TCGA cohort. (A) Circos plot of genome‐wide gene expression. For plots in B–H, left panels show correlation of (B) *GADD45G*, (C) *MTURN*, (D) *TMEM200B*, (E) *RGS20*, (F) *RELL1*, (G) *PGM2*, and (H) *RIPK2* expression (*X*‐axis) with methylation of cg02268510 (*Y*‐axis). Right panels show Kaplan–Meier survival plots of gene expression divided into low and high groups by median value.
**Table S1.** Demographic and clinical characteristics of early‐stage NSCLC patients in TCGA dataset.
**Table S2.** Results for 15 methylation–smoking interactions using a two‐stage association study.
**Table S3.** Smoking‐related characteristics of former and current smokers in early‐stage LUAD.

## References

[mol212482-bib-0001] Anto RJ , Mukhopadhyay A , Shishodia S , Gairola CG and Aggarwal BB (2002) Cigarette smoke condensate activates nuclear transcription factor‐kappaB through phosphorylation and degradation of IkappaB(alpha): correlation with induction of cyclooxygenase‐2. Carcinogenesis 23, 1511–1518. 12189195 10.1093/carcin/23.9.1511

[mol212482-bib-0002] Aran D , Sirota M and Butte AJ (2015) Systematic pan‐cancer analysis of tumour purity. Nat Commun 6, 8971. 26634437 10.1038/ncomms9971 PMC4671203

[mol212482-bib-0003] Athanasios V , Thomais V , Konstantinos F and Spyridon L (2013) Pulmonary oxidative stress, inflammation and cancer: respirable particulate matter, fibrous dusts and ozone as major causes of lung carcinogenesis through reactive oxygen species mechanisms. Int J Environ Res Public Health 10, 3886–3907. 23985773 10.3390/ijerph10093886 PMC3799517

[mol212482-bib-0004] Balduyck B , Sardari NP , Cogen A , Dockx Y , Lauwers P , Hendriks J and Van SP (2011) The effect of smoking cessation on quality of life after lung cancer surgery. Eur J Cardiothorac Surg 40, 1437–1438. 10.1016/j.ejcts.2011.03.004 21498082

[mol212482-bib-0005] Baser S , Shannon VR , Eapen GA , Jimenez CA , Onn A , Lin E and Morice RC (2006) Smoking cessation after diagnosis of lung cancer is associated with a beneficial effect on performance status. Chest 130, 1784–1790. 17166997 10.1378/chest.130.6.1784

[mol212482-bib-0006] Besse B , Adjei A , Baas P , Meldgaard P , Nicolson M , Paz‐Ares L , Reck M , Smit EF , Syrigos K and Stahel R (2014) 2nd ESMO Consensus Conference on Lung Cancer: non‐small‐cell lung cancer first‐line/second and further lines of treatment in advanced disease. Ann Oncol 25, 1475. 24669016 10.1093/annonc/mdu123

[mol212482-bib-0007] Bhatt VR , Batra R , Silberstein PT Jr , Loberiza FR Jr and Ganti AK (2015) Effect of smoking on survival from non‐small cell lung cancer: a retrospective Veterans’ Affairs Central Cancer Registry (VACCR) cohort analysis. Med Oncol 32, 1–6. 10.1007/s12032-014-0339-3 25429831

[mol212482-bib-0008] Bird A (2007) Perceptions of epigenetics. Nature 447, 396. 17522671 10.1038/nature05913

[mol212482-bib-0009] Bjaanæs MM , Fleischer T , Halvorsen AR , Daunay A , Busato F , Solberg S , Jørgensen L , Kure E , Edvardsen H and Børresen‐Dale AL (2016) Genome‐wide DNA methylation analyses in lung adenocarcinomas: association with EGFR, KRAS and TP53 mutation status, gene expression and prognosis. Mol Oncol 10, 330. 26601720 10.1016/j.molonc.2015.10.021 PMC5528958

[mol212482-bib-0010] Chaturvedi MM , Sung B , Yadav VR , Kannappan R and Aggarwal BB (2010) NF‐κB addiction and its role in cancer: ‘one size does not fit all’. Oncogene 30, 1615–1630. 21170083 10.1038/onc.2010.566 PMC3141287

[mol212482-bib-0011] Chen YA , Mathieu L , Sanaa C , Butcher DT , Daria G , Zanke BW , Steven G , Hudson TJ and Rosanna W (2013) Discovery of cross‐reactive probes and polymorphic CpGs in the Illumina Infinium HumanMethylation450 microarray. Epigenetics 8, 203–209. 23314698 10.4161/epi.23470 PMC3592906

[mol212482-bib-0012] Chen SJ , Zhou GB , Zhang XW , Mao JH , De TH and Chen Z (2011) From an old remedy to a magic bullet: molecular mechanisms underlying the therapeutic effects of arsenic in fighting leukemia. Blood 117, 6425–6437. 21422471 10.1182/blood-2010-11-283598 PMC3123014

[mol212482-bib-0013] Chin AI , Dempsey PW , Bruhn K , Miller JF , Xu Y and Cheng G (2002) Involvement of receptor‐interacting protein 2 in innate and adaptive immune responses. Nature 416, 190. 11894097 10.1038/416190a

[mol212482-bib-0014] Crusz SM and Balkwill FR (2015) Inflammation and cancer: advances and new agents. Nat Rev Clin Oncol 12, 584. 26122183 10.1038/nrclinonc.2015.105

[mol212482-bib-0015] Cusick JK , Mustian A , Goldberg K and Reyland ME (2010) RELT induces cellular death in HEK 293 epithelial cells. Cell Immunol 261, 1. 19969290 10.1016/j.cellimm.2009.10.013 PMC3407663

[mol212482-bib-0016] Deponte M (2013) Glutathione catalysis and the reaction mechanisms of glutathione‐dependent enzymes. Biochim Biophys Acta 1830, 3217–3266. 23036594 10.1016/j.bbagen.2012.09.018

[mol212482-bib-0017] Egger G , Liang G , Aparicio A and Jones PA (2004) Epigenetics in human disease and prospects for epigenetic therapy. Nature 429, 457. 15164071 10.1038/nature02625

[mol212482-bib-0018] Feinberg AP and Tycko B (2004) The history of cancer epigenetics. Nat Rev Cancer 4, 143. 14732866 10.1038/nrc1279

[mol212482-bib-0019] Flanders WD , Lally CA , Zhu BP , Henley SJ and Thun MJ (2003) Lung cancer mortality in relation to age, duration of smoking, and daily cigarette consumption: results from Cancer Prevention Study II. Cancer Res 63, 6556. 14559851

[mol212482-bib-0020] Fraga MF and Esteller M (2007) Epigenetics and aging: the targets and the marks. Trends Genet 23, 413–418. 17559965 10.1016/j.tig.2007.05.008

[mol212482-bib-0021] Guo Y , Zhang R , Shen S , Wei Y , Salama SM , Fleischer T , Bjaanaes MM , Karlsson A , Planck M , Su L *et al* (2018) DNA methylation of LRRC3B: a biomarker for survival of early‐stage non‐small cell lung cancer patients. Cancer Epidemiol Biomarkers Prev 27, 1527–1535. 30185536 10.1158/1055-9965.EPI-18-0454 PMC6279565

[mol212482-bib-0022] Guo W , Zhu T , Dong Z , Cui L , Zhang M and Kuang G (2013) Decreased expression and aberrant methylation of Gadd45G is associated with tumor progression and poor prognosis in esophageal squamous cell carcinoma. Clin Exp Metastasis 30, 977–992. 23793925 10.1007/s10585-013-9597-2

[mol212482-bib-0023] Han ZT , Zhu XX , Yang RY , Sun JZ , Tian GF , Liu XJ , Cao GS , Newmark HL , Conney AH and Chang RL (1998) Effect of intravenous infusions of 12‐O‐tetradecanoylphorbol‐13‐acetate (TPA) in patients with myelocytic leukemia: preliminary studies on therapeutic efficacy and toxicity. Proc Natl Acad Sci USA 95, 5357. 9560280 10.1073/pnas.95.9.5357 PMC20265

[mol212482-bib-0024] He L , He T , Farrar S , Ji L , Liu T and Ma X (2017) Antioxidants maintain cellular redox homeostasis by elimination of reactive oxygen species. Cell Physiol Biochem 44, 532–553. 29145191 10.1159/000485089

[mol212482-bib-0025] Hughes JR , Keely J and Naud S (2004) Shape of the relapse curve and long‐term abstinence among untreated smokers. Addiction 99, 29. 14678060 10.1111/j.1360-0443.2004.00540.x

[mol212482-bib-0026] Jaafar R , Mnich K , Dolan S , Hillis J , Almanza A , Logue SE , Samali A and Gorman AM (2018) RIP2 enhances cell survival by activation of NF‐ĸB in triple negative breast cancer cells. Biochem Biophys Res Commun 497, 115–121. 29421659 10.1016/j.bbrc.2018.02.034

[mol212482-bib-0027] Jenifer HD , Bhola S , Kalburgi V , Warad S and Kokatnur VM (2015) The influence of cigarette smoking on blood and salivary super oxide dismutase enzyme levels among smokers and nonsmokers—A cross sectional study. J Tradit Complement Med 5, 100–105. 26151019 10.1016/j.jtcme.2014.11.003 PMC4488049

[mol212482-bib-0028] Karlsson A , Jönsson M , Lauss M , Brunnström H , Jönsson P , Borg A , Jönsson G , Ringnér M , Planck M and Staaf J (2014) Genome‐wide DNA methylation analysis of lung carcinoma reveals one neuroendocrine and four adenocarcinoma epitypes associated with patient outcome. Clin Cancer Res 20, 6127–6140. 25278450 10.1158/1078-0432.CCR-14-1087

[mol212482-bib-0029] Khavari DA , Sen GL and Rinn JL (2010) DNA methylation and epigenetic control of cellular differentiation. Cell Cycle 9, 3880–3883. 20890116 10.4161/cc.9.19.13385

[mol212482-bib-0030] Kooistra MR , Dubé N and Bos JL (2007) Rap1: a key regulator in cell‐cell junction formation. J Cell Sci 120, 17. 17182900 10.1242/jcs.03306

[mol212482-bib-0031] Koshiaris C , Aveyard P , Oke J , Ryan R , Szatkowski L , Stevens R and Farley A (2017) Smoking cessation and survival in lung, upper aero‐digestive tract and bladder cancer: cohort study. Br J Cancer 117, 1224–1232. 28898236 10.1038/bjc.2017.179 PMC5674091

[mol212482-bib-0032] Lacombe L , Fradet V , Levesque E , Pouliot F , Larue H , Bergeron A , Hovington H , Caron A , Nguile‐Makao M , Harvey M *et al* (2016) Phase II drug‐metabolizing polymorphisms and smoking predict recurrence of non‐muscle‐invasive bladder cancer: a gene‐smoking interaction. Cancer Prev Res (Phila) 9, 189–195. 26645279 10.1158/1940-6207.CAPR-15-0069

[mol212482-bib-0033] Lee KWK and Pausova Z (2013) Cigarette smoking and DNA methylation. Front Genet 4, 132. 23882278 10.3389/fgene.2013.00132 PMC3713237

[mol212482-bib-0034] Leung KM , Elashoff RM and Afifi AA (1997) Censoring issues in survival analysis. Annu Rev Public Health 18, 83. 9143713 10.1146/annurev.publhealth.18.1.83

[mol212482-bib-0035] Li Q , Jin W , Cai Y , Yang F , Chen E , Ye D , Wang Q and Guan X (2017) Regulator of G protein signaling 20 correlates with clinicopathological features and prognosis in triple‐negative breast cancer. Biochem Biophys Res Commun 485, 693–697. 28237701 10.1016/j.bbrc.2017.02.106

[mol212482-bib-0036] Liebermann DA , Tront JS , Sha X , Mukherjee K , Mohamedhadley A and Hoffman B (2011) Gadd45 stress sensors in malignancy and leukemia. Crit Rev Oncog 16, 129. 22150313 10.1615/critrevoncog.v16.i1-2.120 PMC3268054

[mol212482-bib-0037] Marabita F , Almgren M , Lindholm ME , Ruhrmann S , Fagerströmbillai F , Jagodic M , Sundberg CJ , Ekström TJ , Teschendorff AE and Tegnér J (2013) An evaluation of analysis pipelines for DNA methylation profiling using the Illumina HumanMethylation450 BeadChip platform. Epigenetics 8, 333. 23422812 10.4161/epi.24008 PMC3669124

[mol212482-bib-0038] Mcnerney ME , Godley LA and Le BM (2017) Therapy‐related myeloid neoplasms: when genetics and environment collide. Nat Rev Cancer 17, 513. 28835720 10.1038/nrc.2017.60 PMC5946699

[mol212482-bib-0039] Mochizuki N , Yamashita S , Kurokawa K , Ohba Y , Nagai T , Miyawaki A and Matsuda M (2001) Spatio‐temporal images of growth‐factor‐induced activation of Ras and Rap1. Nature 411, 1065. 11429608 10.1038/35082594

[mol212482-bib-0040] Moon EY , Lee JH , Lee JW , Song JH and Pyo S (2011) ROS/Epac1‐mediated Rap1/NF‐kappaB activation is required for the expression of BAFF in Raw264.7 murine macrophages. Cell Signal 23, 1479–1488. 21596132 10.1016/j.cellsig.2011.05.001

[mol212482-bib-0041] Nia PS , Weyler J , Colpaert C , Vermeulen P , Marck EV and Schil PV (2005) Prognostic value of smoking status in operated non‐small cell lung cancer. Lung Cancer 47, 351–359. 15713518 10.1016/j.lungcan.2004.08.011

[mol212482-bib-0042] Park ER , Japuntich SJ , Rigotti NA , Traeger L , He Y , Wallace RB , Malin JL , Zallen JP and Keating NL (2012) A snapshot of smokers after lung and colorectal cancer diagnosis. Cancer 118, 3153. 22271645 10.1002/cncr.26545 PMC3342424

[mol212482-bib-0043] Parsons A , Daley A , Begh R and Aveyard P (2010) Influence of smoking cessation after diagnosis of early stage lung cancer on prognosis: systematic review of observational studies with meta‐analysis. BMJ 340, 251. 10.1136/bmj.b5569 PMC2809841 20093278

[mol212482-bib-0044] Remans PHJ , Gringhuis SI , van Laar JM , Sanders ME , Zwartkruis FJT , Levarht EWN , Marcela R , Coffer PJ and Breedveld FC (2004) Rap1 signaling is required for suppression of Ras‐generated reactive oxygen species and protection against oxidative stress in T lymphocytes. J Immunol 173, 920–931. 15240679 10.4049/jimmunol.173.2.920

[mol212482-bib-0045] Sandoval J , Mendez‐Gonzalez J , Nadal E , Chen G , Carmona FJ , Sayols S , Moran S , Heyn H , Vizoso M and Gomez A (2013) A prognostic DNA methylation signature for stage I non‐small‐cell lung cancer. J Clin Oncol 31, 4140. 24081945 10.1200/JCO.2012.48.5516

[mol212482-bib-0046] Schübeler D (2015) Function and information content of DNA methylation. Nature 517, 321. 25592537 10.1038/nature14192

[mol212482-bib-0047] Shen S , Zhang R , Guo Y , Loehrer E , Wei Y , Zhu Y , Yuan Q , Moran S , Fleischer T , Bjaanaes MM *et al* (2018) A multi‐omic study reveals BTG2 as a reliable prognostic marker for early‐stage non‐small cell lung cancer. Mol Oncol 12, 913–924. 29656435 10.1002/1878-0261.12204 PMC5983115

[mol212482-bib-0048] Shui IM , Wong CJ , Zhao S , Kolb S , Ebot EM , Geybels MS , Rubicz R , Wright JL , Lin DW and Klotzle B (2016) Prostate tumor DNA methylation is associated with cigarette smoking and adverse prostate cancer outcomes. Cancer 122, 2168–2177. 27142338 10.1002/cncr.30045 PMC4930391

[mol212482-bib-0049] Siegel RL , Miller KD and Jemal A (2017) Cancer statistics, 2018. CA Cancer J Clin 67, 7. 29313949 10.3322/caac.21442

[mol212482-bib-0050] Slade E and Kraft P (2016) Leveraging methylome‐environment interaction to detect genetic determinants of disease. Hum Hered 81, 26–34. 27490128 10.1159/000447357 PMC5621601

[mol212482-bib-0051] Suk HR , Zhou W , Cogandrew T , Liu G , Su L , Neuberg D , Lynch TJ , Wain JC and Christiani DC (2006) MDM2 polymorphism and recurrence‐free and overall survival in early stage non‐small cell lung cancer (NSCLC). J Clin Oncol 24 (Suppl. 18), 7221.

[mol212482-bib-0052] Sun X , Lu B , Hu B , Xiao W , Li W and Huang Z (2014) Novel function of the chromosome 7 open reading frame 41 gene to promote leukemic megakaryocyte differentiation by modulating TPA‐induced signaling. Blood Cancer J 4, e198. 24681962 10.1038/bcj.2014.18 PMC3972703

[mol212482-bib-0053] Teo H , Ghosh S , Luesch H , Ghosh A , Wong ET , Malik N , Orth A , Jesus PD , Perry AS and Oliver JD (2010) Telomere‐independent Rap1 is an IKK adaptor and regulates NF‐κB‐dependent gene expression. Nat Cell Biol 12, 758–767. 20622870 10.1038/ncb2080

[mol212482-bib-0054] Trerotola M , Relli V , Simeone P and Alberti S (2015) Epigenetic inheritance and the missing heritability. Hum Genomics 9, 17. 26216216 10.1186/s40246-015-0041-3 PMC4517414

[mol212482-bib-0055] Tsurutani J , Castillo SS , Brognard J , Granville CA , Zhang C , Gills JJ , Sayyah J and Dennis PA (2005) Tobacco components stimulate Akt‐dependent proliferation and NFkappaB‐dependent survival in lung cancer cells. Carcinogenesis 26, 1182–1195. 15790591 10.1093/carcin/bgi072

[mol212482-bib-0056] Ueno H , Pradhan S , Schlessel D , Hirasawa H and Sumpio BE (2006) Nicotine enhances human vascular endothelial cell expression of ICAM‐1 and VCAM‐1 via protein kinase C, p38 mitogen‐activated protein kinase, NF‐κB, and AP‐1. Cardiovasc Toxicol 6, 39–50. 16845181 10.1385/ct:6:1:39

[mol212482-bib-0057] Walker MS , Vidrine DJ , Gritz ER , Larsen RJ , Yan Y , Govindan R and Fisher EB (2006) Smoking relapse during the first year after treatment for early‐stage non–small‐cell lung cancer. Cancer Epidemiol Biomarkers Prev 15, 2370. 17132767 10.1158/1055-9965.EPI-06-0509

[mol212482-bib-0058] Walser T , Cui X , Yanagawa J , Lee JM , Heinrich E , Lee G , Sharma S and Dubinett SM (2008) Smoking and lung cancer: the role of inflammation. Proc Am Thorac Soc 5, 811–815. 19017734 10.1513/pats.200809-100TH PMC4080902

[mol212482-bib-0059] Wan ES , Weiliang Q , Andrea B , Carey VJ , Helene B , Rennard SI , Alvar A , Wayne A , Lomas DA and Demeo DL (2012) Cigarette smoking behaviors and time since quitting are associated with differential DNA methylation across the human genome. Hum Mol Genet 21, 3073. 22492999 10.1093/hmg/dds135 PMC3373248

[mol212482-bib-0060] Watt DC , Aitchison TC , Mackie RM and Sirel JM (1996) Survival analysis: the importance of censored observations. Melanoma Res 6, 379–385. 8908598 10.1097/00008390-199610000-00005

[mol212482-bib-0061] Wei Y , Liang J , Zhang R , Guo Y , Shen S , Su L , Lin X , Moran S , Helland Å and Bjaanæs MM (2018) Epigenetic modifications in KDM lysine demethylases associate with survival of early‐stage NSCLC. Clin Epigenetics 10, 41. 29619118 10.1186/s13148-018-0474-3 PMC5879927

[mol212482-bib-0062] Wright J (2013) Epigenetics: reversible tags. Nature 498, 10–11. 10.1038/498S10a 23803942

[mol212482-bib-0063] Yang L , Lee MM , Leung MM and Wong YH (2016) Regulator of G protein signaling 20 enhances cancer cell aggregation, migration, invasion and adhesion. Cell Signal 28, 1663–1672. 27495875 10.1016/j.cellsig.2016.07.017

[mol212482-bib-0064] Ying J , Srivastava G , Hsieh W‐S , Gao Z , Murray P , Liao S‐K , Ambinder R and Tao Q (2005) The stress‐responsive gene GADD45G is a functional tumor suppressor, with its response to environmental stresses frequently disrupted epigenetically in multiple tumors. Clin Cancer Res 11, 6442–6449. 16166418 10.1158/1078-0432.CCR-05-0267

[mol212482-bib-0065] Zaghlool SB , Al‐Shafai M , Al Muftah WA , Kumar P , Falchi M and Suhre K (2015) Association of DNA methylation with age, gender, and smoking in an Arab population. Clin Epigenetics 7, 1–12. 25663950 10.1186/s13148-014-0040-6 PMC4320840

[mol212482-bib-0066] Zhang R , Lai L , He J , Chen C , You D , Duan W , Dong X , Zhu Y , Lin L , Shen S *et al* (2019) EGLN2 DNA methylation and expression interact with HIF1A to affect survival of early‐stage NSCLC. Epigenetics 14, 118–129. 30665327 10.1080/15592294.2019.1573066 PMC6557590

[mol212482-bib-0067] Zhang YL , Wang RC , Cheng K , Ring BZ and Li S (2017) Roles of Rap1 signaling in tumor cell migration and invasion. Cancer Biol Med 14, 90–99. 28443208 10.20892/j.issn.2095-3941.2016.0086 PMC5365179

[mol212482-bib-0068] Zhang N , Wu HJ , Zhang W , Wang J , Wu H and Zheng X (2015) Predicting tumor purity from methylation microarray data. Bioinformatics 31, 3401. 26112293 10.1093/bioinformatics/btv370

[mol212482-bib-0069] Zheng X , Zhang N , Wu HJ and Wu H (2017) Estimating and accounting for tumor purity in the analysis of DNA methylation data from cancer studies. Genome Biol 18, 17. 28122605 10.1186/s13059-016-1143-5 PMC5267453

