# Supplementary material for: SIPA1L3 methylation modifies the benefit of smoking cessation on lung adenocarcinoma survival: an epigenomic–smoking interaction analysis
Source: Mol Oncol. 2019 Apr 17;13(5):1235–48. doi: 10.1002/1878-0261.12482 (PMC6487703; doi:10.1002/1878-0261.12482)

Supplementary Table S1. Demographic and clinical characteristics of early-stage NSCLC patients with both DNA methylation and mRNA expression information in TCGA dataset.

**Characteristic**	LUAD (*N* = 281)	LUSC (*N* = 277)
**Age (years), mean ± SD**	65.39 ± 9.58	67.84 ± 8.85
**Sex, n (%)**		
Female	145 (51.60)	71 (25.63)
Male	136 (48.40)	206 (74.37)
**Smoking cessation, n (%)**		
No	79 (28.11)	87 (31.41)
Yes	192 (68.33)	182 (65.70)
Unknown	10	8
**TNM stage, n (%)**		
I	197 (70.11)	158 (57.04)
II	84 (29.89)	119 (42.96)
**Chemotherapy, n (%)**		
No	95 (33.81)	82 (29.60)
Yes	16 (5.69)	36 (13.00)
Unknown	170	159
**Radiotherapy, n (%)**		
No	106 (37.72)	112 (40.43)
Yes	5 (1.78)	6 (2.17)
Unknown	170	159
**Adjuvant therapy, n (%)**		
No	90 (32.03)	81 (29.24)
Yes	21 (7.47)	37 (13.36)
Unknown	170	159
**Survival year**		
Median (95% CI)	4.38 (3.37–4.93)	5.08 (3.68–6.09)
Censored rate, %	79.36	72.92

NSCLC: non-small cell lung cancer; TGCA: The Cancer Genome Atlas; LUAD: lung adenocarcinoma; LUSC: lung squamous cell carcinomas; 95% CI: 95% confidence interval

Supplementary Table S2. Results for 15 methylation–smoking interactions using a two-stage association study.

Probe	Discovery phase					Validation phase			
	HR	95% CI		*P*	FDR-*q*	HR	95% CI		*P*
**cg02268510**	**1.10**	**1.06**	**1.14**	**4.83E-07**	**0.039**	**1.10**	**1.00**	**1.20**	**0.040**
cg15294851	0.85	0.80	0.91	1.37E-06	0.048	1.12	1.00	1.25	0.052
cg12873707	1.08	1.05	1.12	8.73E-07	0.039	1.05	1.00	1.10	0.058
cg18320188	0.92	0.90	0.96	2.30E-06	0.049	0.95	0.90	1.00	0.064
cg25662053	1.30	1.17	1.45	2.35E-06	0.049	1.16	0.91	1.48	0.224
cg23498273	0.95	0.93	0.97	2.70E-07	0.039	0.99	0.95	1.02	0.447
cg15655316	0.57	0.45	0.72	1.95E-06	0.049	0.83	0.50	1.36	0.457
cg14077144	1.10	1.06	1.14	1.72E-06	0.049	0.98	0.91	1.05	0.579
cg02444961	0.94	0.91	0.96	4.98E-07	0.039	0.99	0.95	1.04	0.754
cg18610079	1.07	1.04	1.10	7.70E-07	0.039	1.01	0.96	1.06	0.812
cg12159302	0.71	0.63	0.82	7.35E-07	0.039	0.97	0.78	1.22	0.821
cg03988119	0.78	0.71	0.86	1.38E-06	0.048	1.09	0.42	2.83	0.852
cg12419067	0.74	0.65	0.83	7.61E-07	0.039	1.01	0.82	1.26	0.891
cg14204281	0.65	0.54	0.77	1.84E-06	0.049	1.02	0.72	1.44	0.916
cg03947979	0.97	0.96	0.98	2.03E-06	0.049	1.00	0.99	1.01	0.971

HR: hazard ratio; 95% CI: 95% confidence interval; FDR: false discovery rate

Supplementary Table S3. Smoking related characteristics of former and current smokers in early-stage LUAD

Variable	Discovery phase					Validation phase	Combined data
	Harvard (*N* = 79)	Spain (*N* = 155)	Norway (*N* = 116)	Sweden (*N* = 63)	Discovery: All (*N* = 413)	TCGA (*N* = 285)	Overall (*N* = 698)
**Age (years), mean ± SD**	67.80 ± 9.07	65.41 ± 10.43	64.89 ± 8.94	63.56 ± 9.96	65.44 ± 9.76	65.30 ± 9.73	65.38 ± 9.74
**Smoking cessation, n (%)**							
No (current smokers)	27 (34.18)	55 (35.48)	42 (36.21)	24 (38.10)	148 (35.84)	81 (28.42)	229 (32.81)
Yes (former smokers)	52 (65.82)	97 (62.58)	74 (63.79)	39 (61.90)	262 (63.44)	194 (68.07)	456 (65.33)
Unknown	0	5	0	0	3	10	13
**Pack-year of smoking**	50.25 ± 35.19	49.62 ± 29.40	30.65 ± 15.45	42.45 ± 16.08	43.23 ± 28.24	41.21 ± 26.87	42.44 ± 27.70
NA	0	12	0	52	64	58	112
**Years of smoking**	50.34 ± 9.84	NA[b]	40.35 ± 11.24	42.67 ± 16.26	44.28 ± 12.08	34.70 ± 13.37	39.75 ± 13.56
NA	0	155	0	48	203	97	300
**Years of smoking cessation[a]**	18.71 ± 13.28	NA[b]	8.47 ± 11.62	18.92 ± 12.57	13.24 ± 13.29	14.43 ± 13.35	13.90 ± 13.32
NA	0	97	0	27	124	23	147

[a] The variable "years of smoking cessation" only analyzed in former smokers;

[b] Years of smoking and years of smoking cessation were not available in Spain center

LUAD: lung adenocarcinoma; TCGA: The Cancer Genome Atlas

Supplementary Figure S1. Quality control procedures for epigenome-wide DNA methylation data.

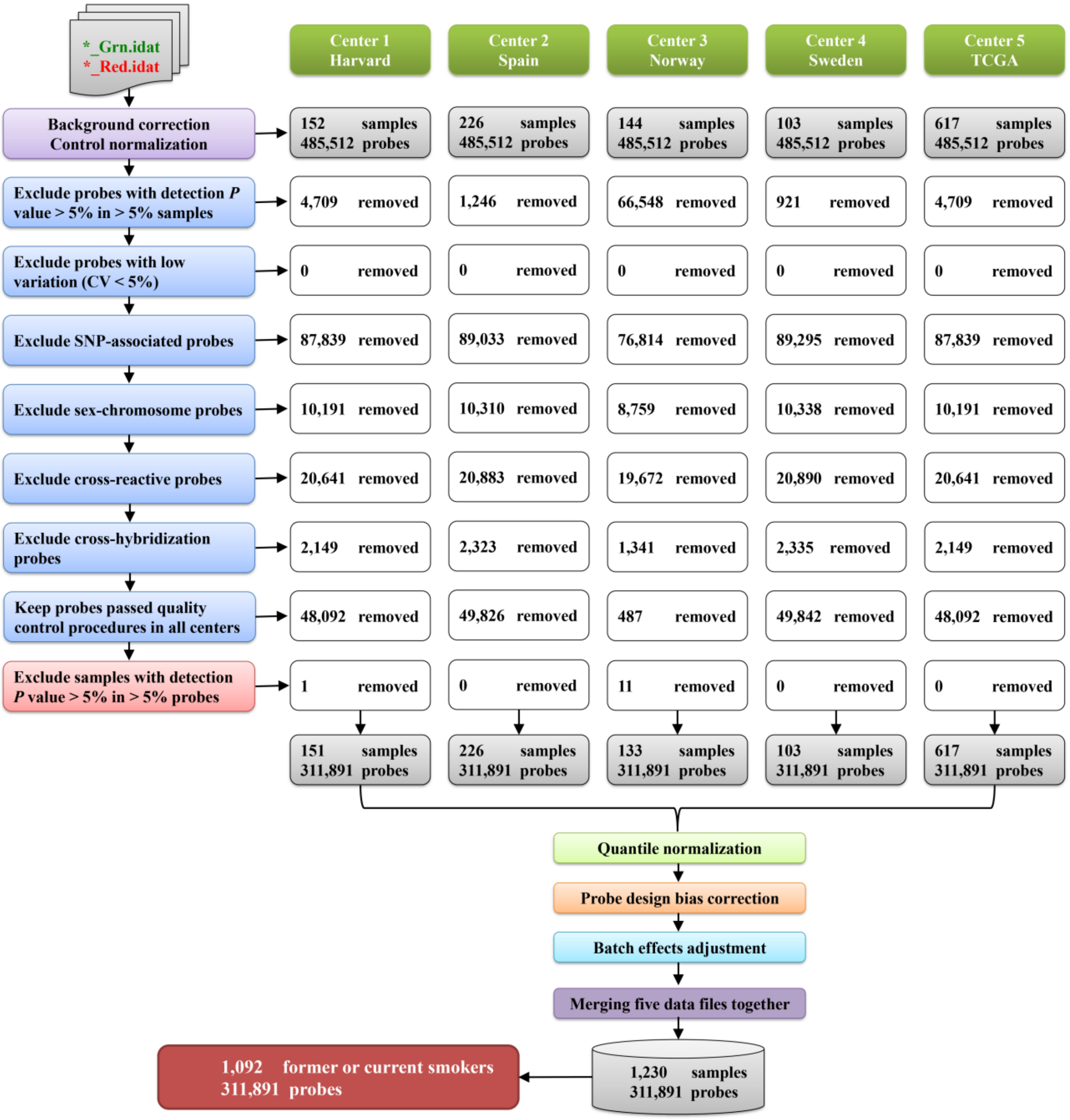

Supplementary Figure S2. Manhattan plot of methylation–smoking interaction *P*-values (A) and DNA methylation main effect *P*-values (B) derived from the histology-stratified Cox proportional hazards model, adjusted for age, sex, smoking status, clinical stage, and study center in the discovery phase. Red line represents FDR-*q* ≤ 0.05. Blue dots above the red line represent 15 CpG probes identified in the discovery phase.

A

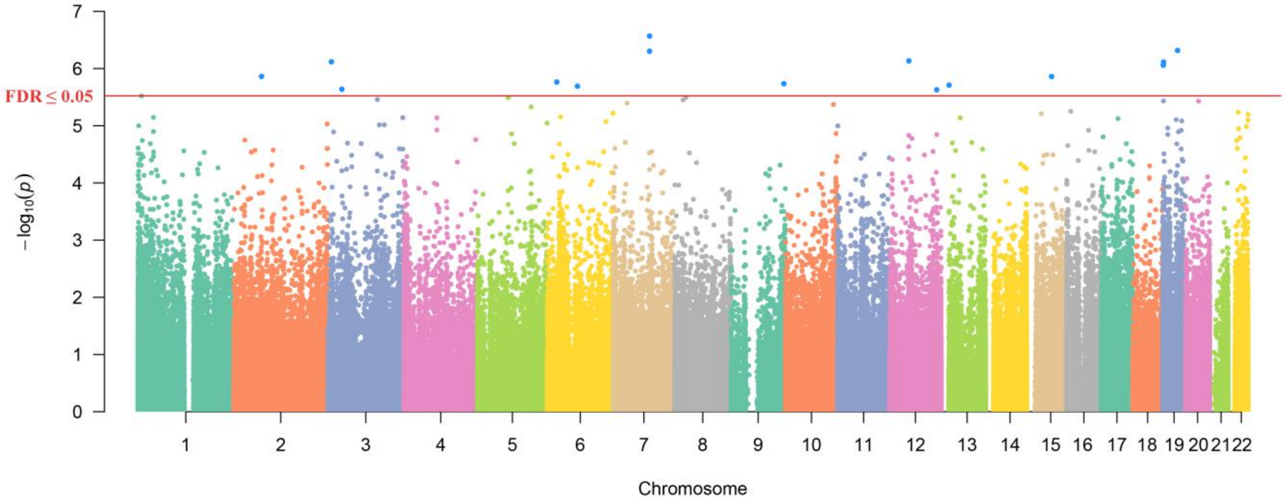

B

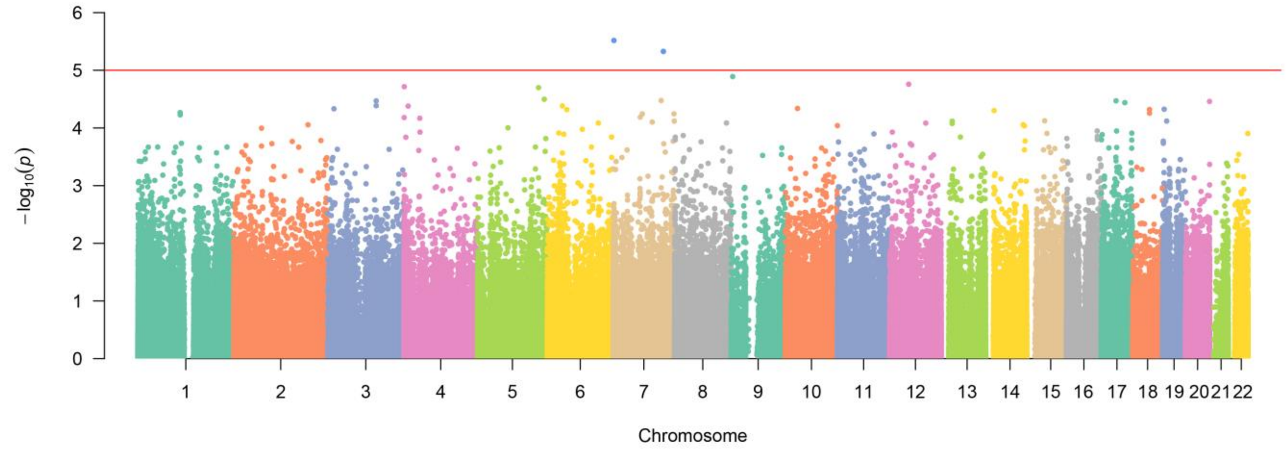

Supplementary Figure S3. Fixed-effect meta-analysis of interaction between DNA methylation of cg02268510 and smoking cessation for LUAD patients from five centers.

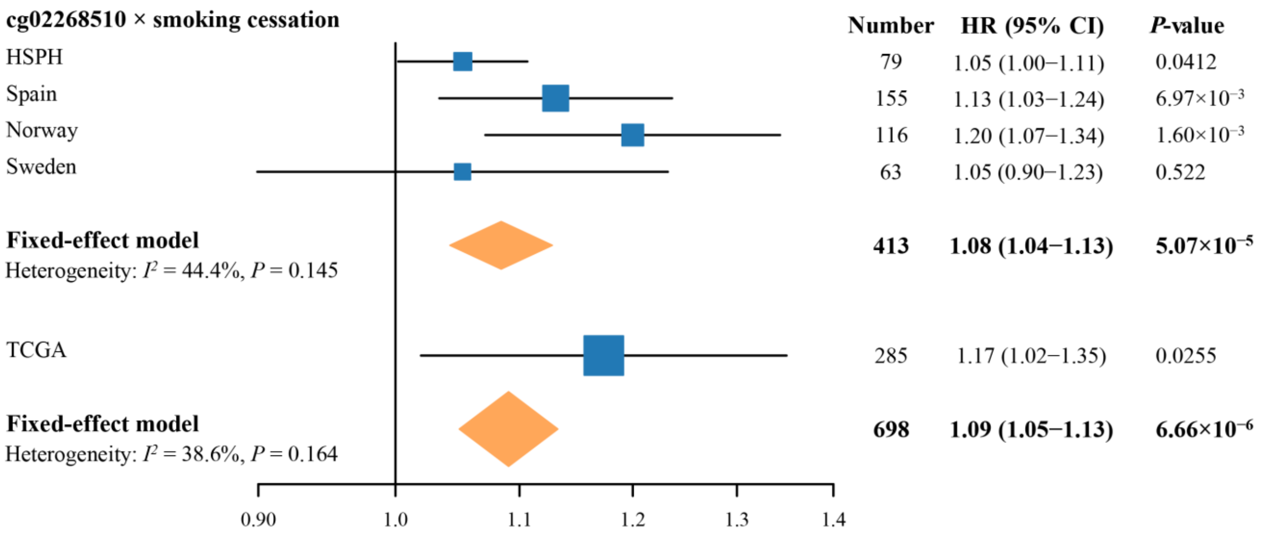

Supplementary Figure S4. Linear regression analysis between methylation of cg02268510 and age (A) as well as smoking related variables: pack-year of smoking (B), year of smoking (C), and year of smoking cessation (D), adjusted for age, sex, smoking status, clinical stage, and study center.

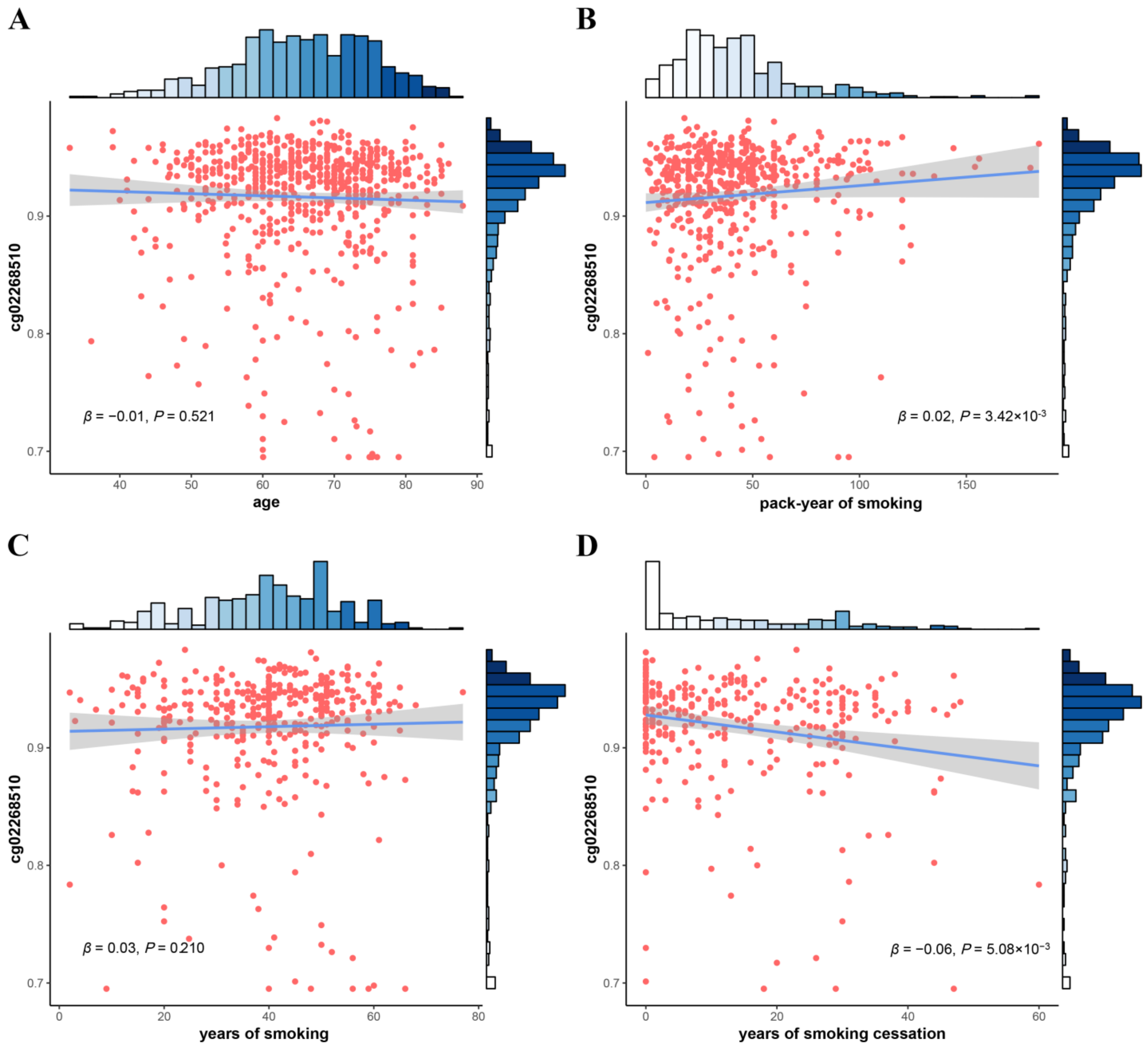

Supplementary Figure S5. Genome-wide methylation transcription analysis from the TCGA cohort. **(A)** Circos plot of genome-wide gene expression. Blue points represent *P*-values of correlation between gene expression and methylation at cg02268510, ordered by genomic position. Grey lines represent significant connections with FDR-*q* ≤ 0.05. For plots **B–H**, left panels show correlation of **(B)** *CADD45G*, **(C)** *MTURN*, **(D)** *TMEM200B*, **(E)** *RGS20*, **(F)** *RELL1*, **(G)** *PGM2*, and **(H)** *RIPK2* expression (*X*-axis) with methylation at cg02268510 (*Y*-axis). β represents coefficient of methylation on gene expression calculated by linear regression. Right panels show Kaplan-Meier survival plots of gene expression divided into low and high groups by median value.

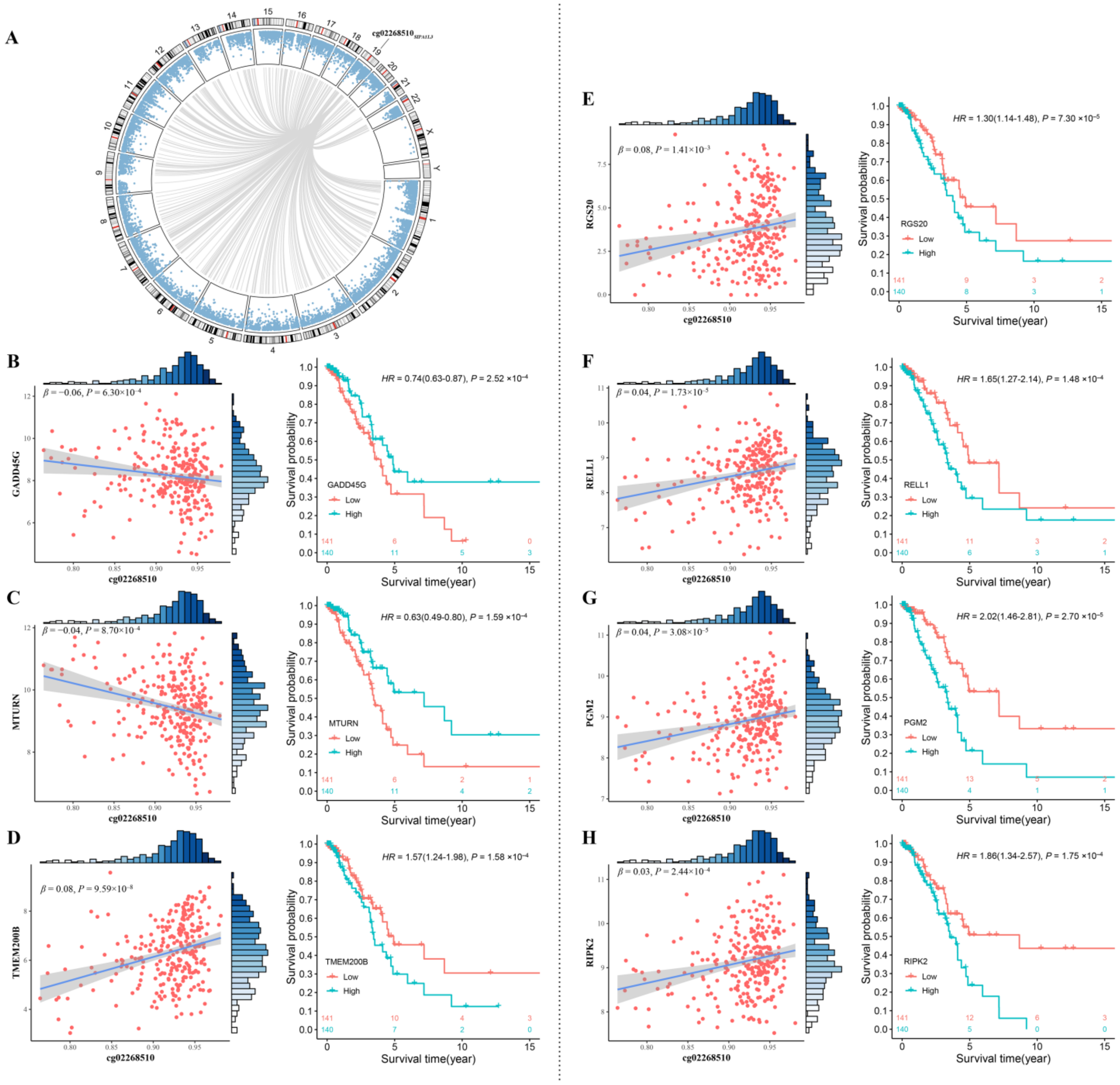

Supplement: Supplementary file 1 — Fig. S1. Quality control processes for DNA methylation chip data. Fig. S2. Manhattan plot of DNA methylation–smoking cessation interaction P‐values (A) and main effect P‐values (B) derived from histology‐stratified Cox proportional hazards model in the discovery phase. Fig. S3. Fixed‐effect meta‐analysis of interaction between DNA methylation of cg02268510 and smoking cessation for LUAD patients from five centers. Fig. S4. Linear regression analysis between methylation of cg02268510 and age (A) as well as smoking‐related variables: pack‐year of smoking (B), year of smoking (C), and year of smoking cessation (D), adjusted for age, sex, smoking status, clinical stage, and study center. Fig. S5. Genome‐wide methylation transcription analysis results from the TCGA cohort. (A) Circos plot of genome‐wide gene expression. For plots in B–H, left panels show correlation of (B) GADD45G, (C) MTURN, (D) TMEM200B, (E) RGS20, (F) RELL1, (G) PGM2, and (H) RIPK2 expression (X‐axis) with methylation of cg02268510 (Y‐axis). Right panels show Kaplan–Meier survival plots of gene expression divided into low and high groups by median value. Table S1. Demographic and clinical characteristics of early‐stage NSCLC patients in TCGA dataset. Table S2. Results for 15 methylation–smoking interactions using a two‐stage association study. Table S3. Smoking‐related characteristics of former and current smokers in early‐stage LUAD. [file MOL2-13-1235-s001.pdf]
